# The Effect of Hot-Air Drying Temperature on the Content of Major Bioactive Constituents and In Vitro Antioxidant Activity of *Sanghuangporus vaninii*

**DOI:** 10.3390/foods15122167

**Published:** 2026-06-16

**Authors:** Jiling Song, Junwen Cheng, Ya Xin, Weidong Yuan, Juanping Jiang

**Affiliations:** 1 Hangzhou Academy of Agricultural Sciences, Hangzhou 310024, China; songjiling860605@163.com (J.S.); xinya0@yeah.net (Y.X.); 2 Zhejiang Academy of Forestry, Hangzhou 310023, China; jwchengzj@163.com; 3 Zhejiang Agricultural Technical Extension Center, Hangzhou 310020, China

**Keywords:** *Sanghuangporus vaninii*, hot-air drying temperature, bioactive constituents, antioxidant activity, correlation analysis

## Abstract

This study investigates the effect of hot-air drying temperature (45–70 °C) on the major bioactive constituents and in vitro antioxidant activity of artificially cultivated *Sanghuangporus vaninii* fruiting bodies. The contents of polyphenols and polysaccharides gradually rose with elevated temperature and peaked at 70 °C, whereas the flavonoid content reached the maximum at 55 °C and declined continuously afterwards. Among the tested bioactive substances, polyphenols exhibited the strongest antioxidant activity, with their DPPH and superoxide anion radical scavenging capacities peaking at 70 °C and hydroxyl radical scavenging capacity peaking at 55 °C. Correlation analysis revealed that the contents of polyphenol and polysaccharide possessed markedly positive correlations with DPPH and superoxide anion scavenging activities, yet no statistically relevant correlation was observed for flavonoids. Cluster analysis classified all tested temperatures into two clusters: low-temperature drying (45–50 °C) yielded inferior bioactive constituents and antioxidant activity, whereas medium-to-high-temperature drying (55–70 °C) delivered superior performance. From a practical perspective, drying at temperatures ≥55 °C is recommended to enhance product quality. Specifically, 70 °C is optimal to maximize overall antioxidant capacity, whereas 55 °C is preferable for flavonoid enrichment and color preservation. These findings provide an evidence-based strategy for the optimization of post-harvest drying processes of *S. vaninii*.

## 1. Introduction

*Sanghuangporus* (Sheng H. Wu, L. W. Zhou & Y. Dai) is a rare, large fungus whose medicinal value has long been documented in classical traditional Chinese medical studies, including Treatise on the Properties of Medicinal Substances and Compendium of Materia Medica [1]. Contemporary pharmacological investigations have validated multiple bioactivities of *Sanghuangporus*, covering anti-tumor [2], antioxidant [3], anti-inflammatory [4], and immunomodulatory effects [5]. These activities are primarily attributed to bioactive components such as polysaccharides, flavonoids, and polyphenols present in its fruiting bodies [6,7,8]. Given the progressive depletion of wild germplasm resources and rising criteria for commodity quality, artificial cultivation has been successfully established for *S. vaninii* and *Sanghuangporus baumii*. Among these two cultivated varieties, *S. vaninii* serves as the primary source for commercial manufacture and market supply due to its short cultivation cycle, high yield, and relatively consistent contents of bioactive constituents. This demonstrates the significant advantage of artificial cultivation in achieving sustainable resource exploitation [9,10]. Despite substantial progress in elite strain breeding and cultivation management techniques [11], the medicinal potency and commercial grading of *Sanghuangporus* remain strongly correlated with post-harvest processing methods. As an indispensable core unit of post-harvest handling, drying functions suppress microbial proliferation, terminate enzymatic reactions, and facilitate storage and transportation [12]. Temperature control during this process is critical, which fundamentally determines the chemical stability of heat-sensitive components (e.g., flavonol aglycones and specified polyphenols) and the structural integrity of cell walls. Inappropriate drying temperatures tend to activate polyphenol oxidase (PPO) and peroxidase (POD), triggering enzymatic browning and polyphenol oxidation polymerisation [13]. Excessively high temperatures may further break the glycosidic bonds of flavonoids and induce structural degradation of flavonoid skeletons. Even relatively stable polysaccharides can undergo chain breakage and conformational rearrangement when exposed to prolonged high temperatures, thereby affecting solubility and immunological activity [14]. Similar studies confirm that high-temperature drying significantly reduces the content of ganoderma triterpenes and chrysanthemum flavonoids [15], confirming the significance of precise temperature control in the processing of medicinal fungi.

Existing research concerning drying technologies for edible and medicinal fungi primarily focuses on individual components or macroscopic properties [16]. Cui et al. [17] systematically investigated the effects of hot-air drying temperature on *Ganoderma* polysaccharides and triterpenoid components, whereas synergistic alterations in antioxidant activity were not elaborated. Similarly, Liu et al. [18] compared the impact of various drying processes on volatile flavor compounds of *Lentinus edodes*, with insufficient attention given to nonvolatile bioactive constituents. The differentiated response mechanisms of active components in *Sanghuangporus* across temperature gradients are not well understood, with a lack of clear, systematic chemical and biological insights. Research predominantly remains at the level of ‘process optimization’ [19,20] and apparent activity ‘differences’ [21]. The deficiency of fundamental theoretical evidence results in experience-dependent drying operations and a lack of unified standardized processing specifications for *Sanghuangporus*, which substantially hinders product quality stability and market competitiveness improvement for *Sanghuangporus* products. To fill this knowledge gap, this current study systematically explores differential responses of three core bioactive constituents (polysaccharides, flavonoids, and polyphenols) to hot-air drying temperatures, ranging from 45 to 70 °C, using artificially cultivated *S. vaninii*. Distinct from prior research limited to individual ingredients or macroscopic drying characteristics, this work evaluates the accumulation patterns of all three constituents under varying temperatures and establishes quantitative correlations with in vitro antioxidant activities, laying foundational data to optimize post-harvest drying processes.

## 2. Materials and Methods

### 2.1. Test Material

Fresh fruiting bodies of artificially cultivated *S. vaninii* were cut into 7 mm-thick slices and dried. Preliminary trials confirmed that drying temperatures below 45 °C resulted in a prolonged drying time (>48 h) and an increased risk of microbial spoilage, whereas temperatures above 70 °C triggered substantial degradation of heat-labile flavonoids and polyphenols. Consequently, six drying temperatures spaced at 5 °C increments (from 45 °C to 70 °C) were selected to cover the practically relevant drying window for this medicinal fungus. Hot-air drying was performed using a DGX-9140 forced-air drying oven (Hangzhou Zhongxu Mechanical Equipment Co., Ltd., Hangzhou, China) fitted with an internal blower and a forced convection circulation; the average airflow across sample surfaces was maintained at 1.5–2.0 m/s. Samples were incubated inside the oven until constant weight was achieved. After cooling, the samples were sealed and stored for later use.

### 2.2. Reagent

Rutin and gallic acid standards were both purchased from Guizhou Dida Technology Co., Ltd., Guiyang, China Sodium hydroxide, sodium nitrite, and ferrous tartrate were all purchased from Aladdin Reagent (Shanghai) Co., Ltd., Shanghai, China Ethanol and petroleum ether were both purchased from Sinopharm Chemical Reagent Co., Ltd., Tianjin, China The assay kits used for measuring DPPH radical scavenging capacity, superoxide anion scavenging capacity, and hydroxyl radical scavenging capacity were purchased from Nanjing Jiancheng Biological Engineering Research Institute, Nanjing, China.

### 2.3. Equipment and Instruments

The following equipment and instruments were used: high-speed universal pulverizer (FW, Hangzhou Xuzhong Machinery Equipment Co., Ltd., Hangzhou, China), freeze dryer (Labconco, Waltham, MA, USA), multifunctional high-speed centrifuge (CL31R, Thermo Fisher Scientific, Hillsboro, OR, USA), rotary evaporator (R-210, Buchi, Flawil, Switzerland), thermostatic drying oven (DGX-9140, Hangzhou Xuzhong Machinery Equipment Co., Ltd., Hangzhou, China), microplate reader (Infinite M1000 Pro, Tecan, Männedorf, Switzerland), scanning electron microscope (Gemini SEM300, Carl Zeiss (Shanghai) Management Co., Ltd., Shanghai, China), and texture analyser (FTC TMS-Plus, Zhejiang TopCloud Agricultural Technology Co. Ltd., Hangzhou, China).

### 2.4. Indicator Measurement

#### 2.4.1. Drying Properties

##### Dry-Basis Moisture Content

The dry-basis moisture content (St) of the *S. vaninii* sample is calculated using the following formula:

(1) St = (Qt − Q)/Q

where St denotes the moisture content of the sample on a dry basis at time t (g/g); Qt denotes the sample weight measured at drying time t (g); and Q denotes the weight of the sample dried to a constant weight (g).

##### Drying Rate

The drying rate (DR) of the *S. vaninii* sample is calculated based on the variation in dry-basis moisture content between two adjacent sampling moments using the following formula:

(2) *DR* = (*X_t_*_1_ − *X_t_*_2_)/(*t*_2_ − *t*_1_)

where DR is the average drying rate of the sample (g·g^−1^·h^−1^); X_t1_ is the dry-basis moisture content at time t_1_ (g/g); X_t2_ is the dry-basis moisture content at time t_2_ (g/g).

#### 2.4.2. Color

Color determination was performed following the experimental protocol reported by Wei Mengxiang et al. [22] via a color difference meter. Fresh *S. vaninii* slices were used as the blank control, and color parameters (*L*_0_, *a*_0_, and *b*_0_) were detected at the central region of the slices. The total color difference (ΔE) after drying was calculated using the following formula:

$$\Delta E=\;\sqrt{{\left(L_{0}\;-\;L\right)}^{2}+{\left(a_{0}\;-\;a\right)}^{2}+{\left(b_{0}\;-\;b\right)}^{2}}$$

where Δ*E* represents the total color difference value before and after drying. *L*_0_, *a*_0_, and *b*_0_ denote the lightness, red-green chromaticity, and yellow-blue chromaticity of the fresh sample, respectively. L, a, and b denote the lightness, red-green chromaticity, and yellow-blue chromaticity of the dried sample, respectively.

#### 2.4.3. Texture

The texture properties of fresh and dried *S. vaninii* fruiting bodies were measured using a texture analyzer (FTC TMS-Plus, Zhejiang TopCloud Agricultural Technology Co. Ltd., Hangzhou, China) fitted with a 5 mm-diameter cylindrical probe. The test parameters were set as follows: pre-test speed of 1 mm/s; test speed of 1 mm/s; post-test speed of 1 mm/s. Compression deformation was fixed at 20% of each sample’s initial height with a trigger force of 0.05 N and an interval of 3 s between compressions. Ten replicate specimens were analyzed per drying treatment.

#### 2.4.4. Microstructure

Dried *S. vaninii* samples were cut into cubic blocks with dimensions of 4 mm × 4 mm × 4 mm. The microstructure of the sample surfaces was observed using a scanning electron microscope at 1000× magnification.

#### 2.4.5. Determination of Active Compounds in *S. vaninii*

The phenol-sulfuric acid method was adopted to quantify the polysaccharide content; the sodium nitrite-aluminum nitrate colorimetric assay was used for flavonoid quantification; ferrous tartrate colorimetry was applied to determine the total polyphenol content. Standard curves were obtained by plotting the mass concentration of the standard substance on the *x*-axis and the OD value on the *y*-axis. The corresponding regression equations were calculated as follows: *y* = 2.812*x* + 0.301 (*R*^2^ = 0.970); *y* = 0.09831*x* − 0.00475 (*R*^2^ = 0.99911); and *y* = 182.3958*x* + 2.2143 (*R*^2^ = 0.9981). Sample absorbance was measured at 490 nm, 510 nm, and 540 nm to calculate the polysaccharide, flavonoid, and polyphenol contents.

#### 2.4.6. Determination of Antioxidant Activity

##### DPPH Radical Scavenging Capacity

DPPH radical scavenging activity was measured using a commercial assay kit (Suzhou keming, Suzhou, China). The assay is based on the reduction of DPPH (stable free radical) by antioxidants, which lowers the absorbance values at 515 nm. In short, 20 μL of sample solution was blended with 380 μL of DPPH working solution, kept in the dark at ambient temperature for 20 min, and absorbance was measured at 515 nm. The scavenging rate was calculated as follows: Scavenging rate (%) = (A_blank − A_sample)/A_blank × 100% [23].

##### Superoxide Anion Radical Scavenging Capacity

The superoxide anion radical scavenging activity was measured using a commercial kit (Suzhou keming, Suzhou, China). The assay principle is as follows: superoxide anions generated by the AP-TEMED system react with hydroxylamine to produce NO_2_^−^, which then forms a red azo derivative upon reacting with p-aminobenzenesulfonic acid and α-naphthylamine (530 nm). The reaction mixture contained 10 μL of reagent one, 40 μL of reagent two, 25 μL of H_2_O, and 25 μL of the sample, incubated at 25 °C for 1 min, followed by the addition of 50 μL of reagent three and incubation at 37 °C for 30 min. Afterwards, 50 μL of reagent four and 50 μL of reagent five were added, and the mixture was incubated at 37 °C for 20 min for color development. Absorbance was measured at 530 nm. The scavenging rate was calculated as follows: Scavenging rate (%) = (A_control − A_sample)/A_control × 100% [23].

##### Hydroxyl Radical Scavenging Capacity Assay

The hydroxyl radical scavenging activity was measured using a commercial kit (Suzhou keming, Suzhou, China). The assay is based on the Fenton reaction (H_2_O_2_/Fe^2+^) to produce hydroxyl radicals, which react with salicylic acid to form a colored product (510 nm). The reaction mixture contained 100 μL of reagent one, 100 μL of reagent two, 300 μL of H_2_O, 200 μL of the sample, and 200 μL of reagent three, followed by incubation at 37 °C for 20 min. Absorbance was measured at 510 nm. A control group (sample replaced with H_2_O) and a blank group (reagent two replaced with H_2_O) were included. The scavenging rate was calculated as follows: Scavenging rate (%) = (A_control − A_sample)/(A_control − A_blank) × 100% [23].

### 2.5. Statistical Analysis

All the results were presented as the mean ± standard deviation (SD). Statistical computations were completed with SPSS 17.0 software (SPSS Inc., Chicago, IL, USA). One-way analysis of variance (ANOVA) was adopted to compare measurement indicators across distinct drying temperature treatments. When a significant difference was detected (*p* < 0.05), Tukey’s honestly significant difference (HSD) post hoc test was applied for multiple pairwise comparisons. Statistical significance was defined at *p* < 0.05, while *p* < 0.01 indicated highly significant differences. Correlation analysis between active component contents and antioxidant activity was performed using Pearson’s correlation coefficient. Graphs were generated using Origin 2021 (OriginLab Corp., Northampton, MA, USA).

## 3. Results

### 3.1. Effects of Hot-Air Drying Temperature on Dry-Base Moisture Content and Drying Rate of S. vaninii Fruiting Bodies

**Figure 1:**
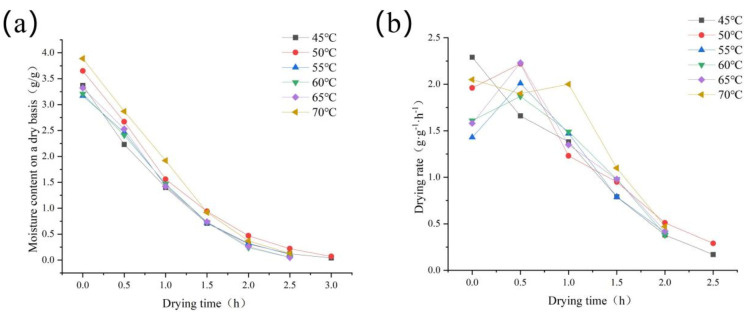
Effects of hot-air drying temperature on dry-basis moisture content and drying rate of *S. vaninii* fruiting bodies: (**a**) dry-basis moisture content variation curve; (**b**) drying rate variation curve.

Dry-base moisture content and drying rate are key indicators for evaluating drying efficiency. As illustrated in Figure 1a, the dry-basis moisture content of *S. vaninii* fruiting bodies declines continuously with increasing drying duration during hot-air drying. At the initial drying stage (0–0.5 h), the moisture content decreased rapidly across all treatments, with the most significant reduction detected under the 65 °C treatment. After 2.5 h of drying, the dry-basis moisture content at 60 °C and 65 °C had decreased to 0.05 g/g for the 60 °C and 65 °C groups, whereas values reached 0.12 g/g and 0.22 g/g at 45 °C and 50 °C, respectively. This indicates that higher drying temperatures markedly shorten the drying duration. Throughout the drying process, the moisture content at 50 °C remained consistently higher than that of the remaining temperature groups, implying unsatisfied rapid water migration under such temperatures.

The variation in drying rate further confirms the critical regulatory role of temperature. As illustrated in Figure 1b, within the initial drying phase (0–0.5 h), the 50 °C and 65 °C treatments exhibit superior initial drying rates of 2.22 g·g^−1^·h^−1^ and 2.23 g·g^−1^·h^−1^, respectively. Overall, the drying rate gradually decreased with increasing drying time, which is consistent with the characteristics of a typical drying curve. At identical time points, higher temperatures corresponded to higher drying rates; notably, the 70 °C group sustained a higher drying rate than all other groups after 1.5 h. This suggests that increasing the drying temperature effectively accelerates the diffusion and evaporation of internal moisture, thereby enhancing drying efficiency.

### 3.2. Effects of Hot-Air Drying Temperature on the Color of S. vaninii Fruiting Bodies

**Figure 2:**
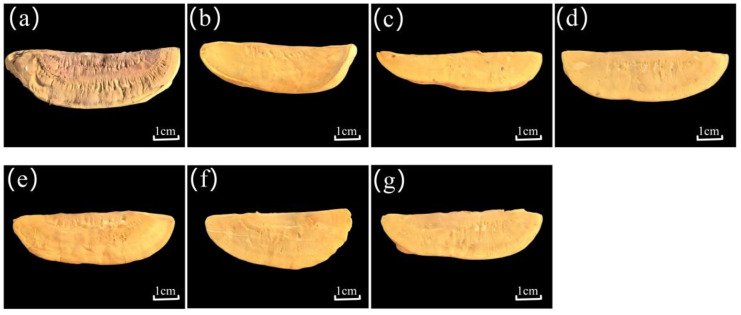
Samples of *S. vaninii* fruiting bodies under different hot-air drying temperature conditions: (**a**) Fresh; (**b**) 45 °C; (**c**) 50 °C; (**d**) 55 °C; (**e**) 60 °C; (**f**) 65 °C; (**g**) 70 °C.

Color serves as a vital indicator to evaluate the external quality of dried products. As shown in Table 1 and Figure 2, hot-air drying temperature exerted significant influences on the color parameters (*L*, a, and b) and total color difference (Δ*E*) of *S. vaninii* fruiting bodies (*p* < 0.05). Compared to fresh samples, all drying treatments significantly increased the lightness value (*L*) (*p* < 0.05). The maximum L values were recorded at 55 °C (62.94) and 60 °C (62.97), without statistical discrepancies relative to the remaining drying treatments. The a-value (redness) showed relatively minor overall changes, and only the 50 °C treatment group (7.78) possessed significantly higher values than the fresh sample (7.62) and the 70 °C group (7.38). The b value (yellowness) exhibited more pronounced variations, with the peak value (65.46) observed at 55 °C, which was significantly higher than the fresh sample (60.73), and 45 °C and 60 °C treatments.

The total color difference (Δ*E*) was adopted for comprehensive quantification of the overall color deviation between dried samples and fresh counterparts. As shown in Table 1, the most prominent color shift occurred under the 55 °C treatment (Δ*E* = 5.55), which was statistically superior to all other tested groups (*p* < 0.05). The ΔE values of the residual treatment groups ranged from 3.9 to 4.39, among which no remarkable intergroup difference was detected. These results suggest that drying at 55 °C may stimulate color-related biochemical reactions (e.g., the Maillard reaction or phenolic oxidation), eventually triggering the most significant color changes.

### 3.3. Effects of Hot-Air Drying Temperature on Texture Properties of S. vaninii

Table 2 shows that the drying temperature induced significant variations in all texture parameters of *S. vaninii* fruiting bodies (*p* < 0.05). Hardness displayed an initial increase, followed by a descending trend along with an elevated drying temperature. Fresh samples demonstrated the minimum hardness (64.75 N), which significantly increased after drying treatment and peaked at 55 °C (274.00 N) and 60 °C (274.90 N), which were significantly higher than those of all remaining treatments. Although hardness decreased slightly at 65 °C and 70 °C, it remained significantly higher than at 45 °C and 50 °C. This suggests that drying conditions between 55 °C and 60 °C most effectively induce tissue shrinkage and hardening.

Elasticity fluctuated within a narrow range, from 3.70 to 4.18. The maximum elasticity of 4.18 was obtained under the 45 °C treatment, whereas the minimum value (3.70) was detected at 50 °C. Cohesiveness reached the highest level of 0.46 in fresh samples and generally decreased after drying. Cohesiveness was relatively well maintained at 45 °C and 55 °C (0.45 and 0.44, respectively), while other treatments showed significant reductions.

Adhesiveness and chewiness exhibited similar trends, and both parameters attained their maximum values at 55 °C, reaching 124.84 N and 478.09 N·mm, respectively. These values were significantly higher than those of the other treatments (*p* < 0.05). Such results implied that 55 °C served as the critical temperature for the formation of the toughest and most chew-resistant texture in *S. vaninii* fruiting bodies. However, excessively high drying temperatures (e.g., 70 °C) resulted in reduced adhesiveness and chewiness, potentially due to temperature-induced damage or collapse of tissue structures.

### 3.4. Effects of Hot-Air Drying Temperature on the Microstructure of S. vaninii Fruiting Bodies

**Figure 3:**
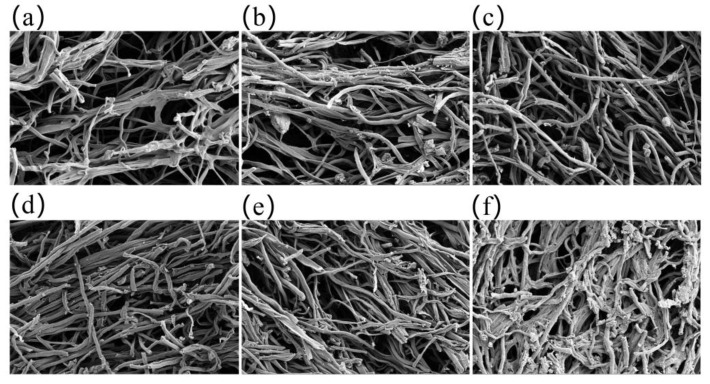
Effects of hot-air drying temperatures on the microscopic structure of *S. vaninii* fruiting bodies: (**a**) 45 °C; (**b**) 50 °C; (**c**) 55 °C; (**d**) 60 °C; (**e**) 65 °C; (**f**) 70 °C.

As displayed in Figure 3, different hot-air drying temperatures exert remarkable impacts on the microstructure of *S. vaninii*. Scanning electron microscopy (SEM) observations reveal that different drying temperatures markedly affect the microstructure of *S. vaninii* fruiting bodies. As shown in Figure 3a,b, samples dried at 45 °C and 50 °C possessed relatively smooth epidermal surfaces with intact cellular structures arranged into loose and porous reticular frameworks. However, when the temperature rose to 55 °C, microscopic pores commenced shrinkage that was accompanied by mild surface wrinkling in partial tissue regions (Figure 3c). Figure 3d–f show that, as the drying temperature increased further, the cellular structures underwent pronounced changes: the surface wrinkled more intensely, the pore structures diminished substantially, and the tissue densified. Notable structural collapse and cell fusion were particularly visualized in the 70 °C treatment group. These results suggest that higher drying temperatures accelerate the shrinkage and collapse of cell walls in fruiting bodies and facilitate microstructure compaction. This may significantly impact the fruiting body’s textural properties, as well as the dissolution and retention of bioactive constituents.

### 3.5. Effect of Hot-Air Drying Temperature on the Contents of Bioactive Component

**Figure 4:**
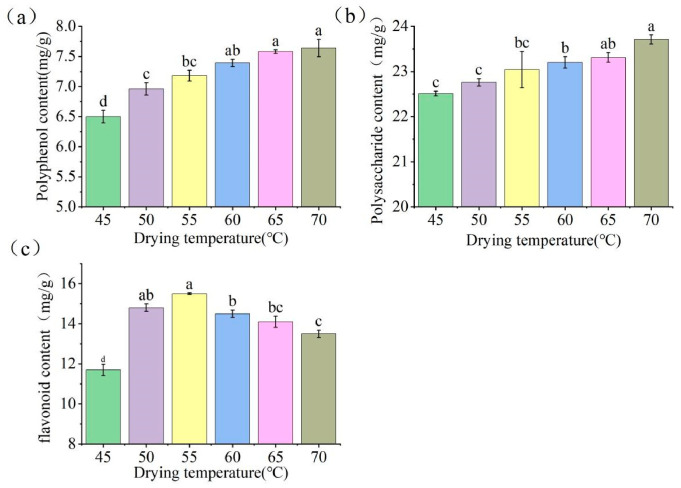
Effects of hot-air drying temperatures on polyphenol, polysaccharide, and flavonoid contents: (**a**) polyphenols; (**b**) polysaccharides; (**c**) flavonoids. Different letters indicate significant differences (*p* < 0.05).

All three active components in *S. vaninii* fruiting bodies exhibited distinct temperature-dependent variations under varied hot-air drying temperatures. As shown in Figure 4a, polyphenol content increased gradually with rising drying temperature and peaked at 70 °C with a value of 7.63 mg/g. Figure 4b shows that polysaccharide content underwent a gradual ascending trend alongside thermal elevation, reaching the maximum of 23.71 mg/g at 70 °C, with significant differences detected across all treatments. Flavonoid content was susceptible to thermal variations; its concentration rose initially and declined subsequently with rising drying temperatures (see Figure 4c), reaching the maximum content of 15.5 mg/g at 55 °C.

### 3.6. Effects of Hot-Air Drying Temperatures on Polyphenol Antioxidant Activity

**Figure 5:**
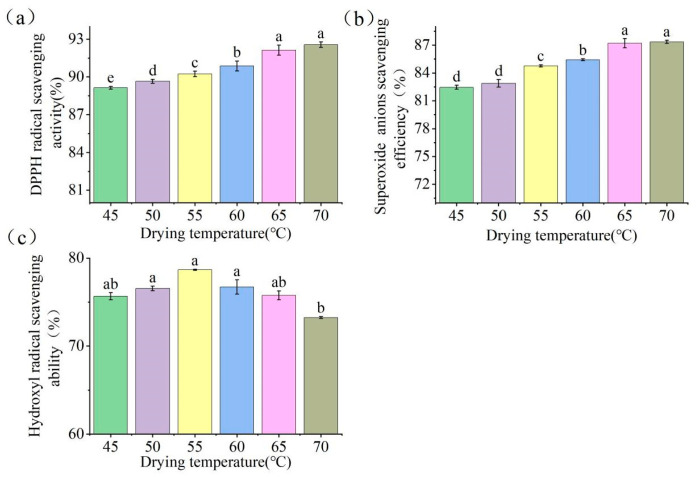
Effects of hot-air drying temperatures on the antioxidant activity of polyphenols: (**a**) DPPH radical scavenging ability; (**b**) superoxide anion scavenging ability; (**c**) hydroxyl radical scavenging ability. Different letters indicate significant differences (*p* < 0.05).

Hot-air drying temperature imposed significant regulatory effects on the antioxidant performance of polyphenols from *S. vaninii* fruiting bodies. As illustrated in Figure 5a,b, the DPPH radical and superoxide anion radical scavenging capacities of polyphenols progressively improved as the drying temperature increased. The minimum scavenging efficiencies of the two indicators were detected at 45 °C (89.1% and 82.27%, respectively), whereas their maximal values were obtained at 70 °C (93.15% and 87.27%), with no statistically meaningful divergence relative to the 65 °C group. Regarding hydroxyl radical scavenging capacity (see Figure 5c), there was an initial increase followed by a decrease as the temperature rose, peaking at 55 °C with a scavenging rate of 78.66%. This optimum value showed no remarkable difference versus the 45 °C, 50 °C, 60 °C, and 65 °C treatments, but was statistically superior to that of the 70 °C group (*p* < 0.05). Comprehensive comparison indicates the following order of radical scavenging potency: DPPH radical > superoxide anion radical > hydroxyl radical.

### 3.7. Effects of Hot-Air Drying Temperatures on Polysaccharide Antioxidant Activity

**Figure 6:**
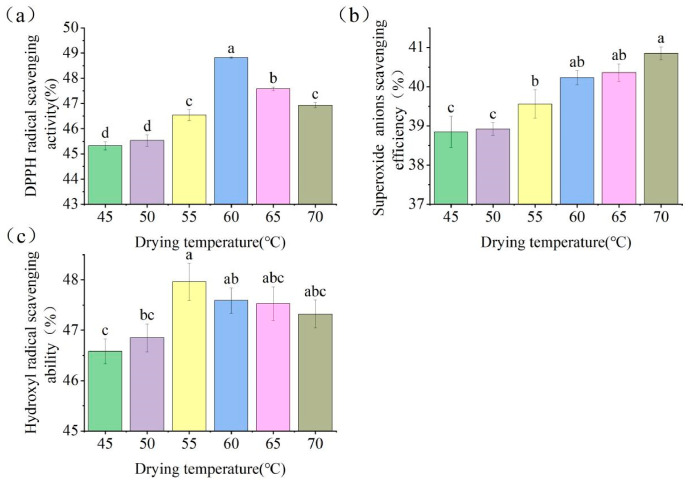
Effect of hot-air drying temperature on the antioxidant activity of polysaccharides: (**a**) DPPH radical scavenging ability; (**b**) superoxide anion scavenging ability; (**c**) hydroxyl radical scavenging ability. Different letters indicate significant differences (*p* < 0.05).

Hot-air drying temperature exerted remarkable impacts on the antioxidant properties of polysaccharides from *S. vaninii* fruiting bodies. As illustrated in Figure 6a, the DPPH radical scavenging capacity of polysaccharides initially increased and subsequently decreased with the increment of drying temperature, reaching the peak value of 48.82% at 60 °C, which was significantly higher than all other treatments (*p* < 0.05). Regarding superoxide anion radical scavenging capacity (Figure 6b), the 70 °C treatment exhibited the highest scavenging rate of 40.85%. This value had no statistical difference compared with the 60 °C and 65 °C treatments, but was significantly higher than those of the 55 °C, 50 °C, and 45 °C treatments (*p* < 0.05). Regarding hydroxyl radical scavenging capacity (Figure 6c), the 55 °C treatment exhibited the highest scavenging rate (47.96%), showing significant differences compared to all remaining experimental groups (*p* < 0.05).

A comprehensive analysis revealed the radical scavenging potency sequence of polysaccharides as follows: DPPH radical > hydroxyl radical > superoxide anion radical. Furthermore, polysaccharides exhibited significantly weaker antioxidant activity than polyphenols, demonstrating that polyphenols served as the primary contributors to the overall antioxidant activity of *S. vaninii* fruiting bodies across different drying temperatures.

### 3.8. Effects of Hot-Air Drying Temperatures on Flavonoid Antioxidant Activity

**Figure 7:**
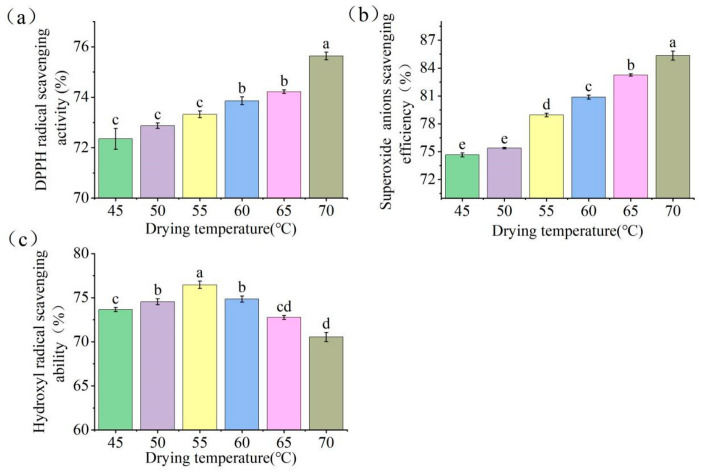
Effects of hot-air drying temperatures on the antioxidant activity of flavonoids: (**a**) DPPH radical scavenging ability; (**b**) superoxide anion scavenging ability; (**c**) hydroxyl radical scavenging ability. Different letters indicate significant differences (*p* < 0.05).

Different drying temperatures exerted significant effects on the antioxidant activity of flavonoids from *S. vaninii* fruiting bodies. As illustrated in Figure 7a,b, the flavonoids’ scavenging capacity for both DPPH and superoxide anion radicals increased gradually with rising temperature. The minimum scavenging rates for both radicals were recorded at 45 °C (72.35% and 74.65%, respectively), whereas the maximum values were obtained at 70 °C (75.64% and 85.34%, respectively), which differed significantly from all remaining experimental groups (*p* < 0.05). In terms of hydroxyl radical scavenging capacity (see Figure 7c), there was an initial increase followed by a decrease in capacity with rising temperature. The strongest capacity was observed at 55 °C (76.45%), which also exhibited significant differences compared to the other treatments (*p* < 0.05).

A comprehensive analysis defined the potency ranking of flavonoids toward three radicals as follows: superoxide anion radical > hydroxyl radical > DPPH radical. Furthermore, the overall antioxidant activity of flavonoids was superior to that of polysaccharides, yet inferior to polyphenols.

### 3.9. Analysis of Bioactive Constituents in S. vaninii Fruiting Bodies and Their Correlation with Antioxidant Activity

The Pearson correlation analysis results (see Table 3) revealed statistically significant correlations between the contents of bioactive compounds and corresponding antioxidant indicators in *S. vaninii* fruiting bodies subjected to different drying temperatures. Specifically, both polyphenols and polysaccharides exhibited extremely significant positive correlations with DPPH radical and superoxide anion scavenging capacities (polyphenols: *r* = 0.96 and 0.96, *p* < 0.01; polysaccharides: *r* = 0.95 and 0.97, *p* < 0.01). Additionally, DPPH radical scavenging capacity was strongly and positively correlated with superoxide anion scavenging capacity (*r* = 0.98, *p* < 0.01). Among the three bioactive components, an extremely significant positive correlation was detected between polyphenols and polysaccharides (*r* = 0.95, *p* < 0.01), whereas no significant correlation was observed between flavonoids and the other two constituents. Furthermore, flavonoids displayed an insignificant association with all determined antioxidant parameters. These results suggest that hot-air drying temperature primarily influences the overall antioxidant capacity of *S. vaninii* fruiting bodies by regulating the accumulation of polyphenols and polysaccharides.

### 3.10. Cluster Analysis of Different Hot-Air Drying Temperatures

**Figure 8:**
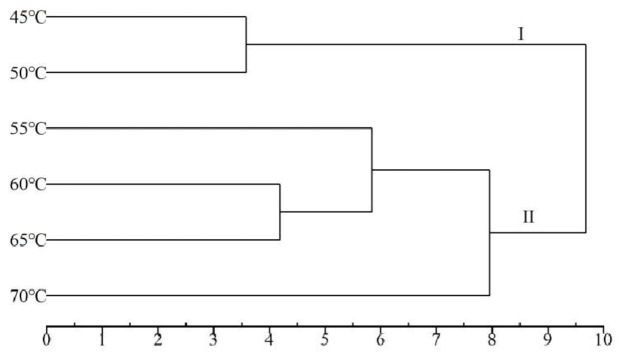
Cluster analysis based on active ingredient contents and antioxidant activity.

Based on the results of the systematic cluster analysis (see Figure 8), the six hot-air drying temperatures were classified into two distinct clusters at the squared Euclidean distance threshold of 8. Cluster I covered two low-temperature treatments at 45 °C and 50 °C, characterized by generally inferior contents of bioactive constituents and weaker antioxidant activity. Specifically, the polyphenol and polysaccharide contents reached the minimum values alongside relatively low flavonoid concentrations. Regarding antioxidant activity, the scavenging capacity for all three types of free radicals was significantly lower than that in Cluster II. Cluster II comprises four moderate-to-high-temperature drying treatments (55 °C, 60 °C, 65 °C, and 70 °C), presenting completely opposite physicochemical characteristics compared with Cluster I.

This group generally exhibits a higher bioactive ingredient content, in which polyphenol and polysaccharide contents peaked at 70 °C. In terms of antioxidant activity, hydroxyl radical scavenging capacity achieved maximum at 55 °C; however, both DPPH radical and superoxide anion scavenging capacities rose continuously with increasing temperature, reaching their highest levels at 70 °C. Notably, an extremely significant positive correlation (*r* = 0.95) was detected between polyphenol and polysaccharide contents in Cluster II., and both constituents exhibited prominent positive correlations with the two primary radical scavenging indicators, consistent with the results of the overall correlation analysis. Collectively, cluster results verified that drying at or above 55 °C facilitates the accumulation of bioactive compounds and improvement of antioxidant activity. This provides theoretical support for determining the optimal drying process for *S. vaninii* fruiting bodies.

## 4. Discussion

### 4.1. Effects of Hot-Air Drying Temperature on the Accumulation of Bioactive Constituents in S. vaninii Fruiting Bodies

The drying temperature serves as one of the core determinants governing the retention of bioactive substances and the final pharmacological efficacy of Chinese herbal medicines [24,25]. This study systematically revealed the regulatory effects of hot-air drying temperature on the physical properties, accumulation of active components, and antioxidant activity of *S. vaninii* fruiting bodies, as well as divergent underlying mechanisms. The findings indicate that the content of polyphenols progressively increases with rising drying temperatures (45–70 °C), peaking at 70 °C. This could be related to the preservation of polyphenol content and active groups at higher temperatures, yet relevant causation needs further verification [26,27,28]. Meanwhile, elevated temperatures also significantly accelerate water migration and vaporization, thereby shortening the duration of samples retained at moderate moisture content (where enzyme activity is higher), which may mitigate enzymatic loss of polyphenols. These findings are consistent with previous research from Choi et al. [29], who verified that high-temperature short-time drying enhances the polyphenol content in *Lentinus edodes*. In contrast, at lower temperatures, drying at 45–50 °C causes limited thermal damage yet prolongs the total drying duration, aggravating polyphenol consumption via enzymatic oxidation [27]. Polysaccharides, being relatively thermostable macromolecular polymers [30], also exhibited increased content with rising temperature, peaking at 70 °C. It is inferred that, under the hot-air drying conditions applied in this experiment, polysaccharide structures remained largely intact. High temperatures remodel or rupture cell wall architecture and thereby promote the release of intracellular polysaccharides. Additionally, it may also suppress microbial proliferation and hydrolase activity, curbing polysaccharide breakdown [31]. Distinct from polyphenols and polysaccharides, the content of flavonoids exhibited an initial increase followed by a decrease with rising temperature, peaking at 55 °C. At this temperature, enzyme activity was effectively inhibited, while cell wall softening and cracking favored the dissolution and extraction of flavonoids [32]. However, when temperatures rise to 60 °C and above (especially at 70 °C), sustained thermal stress may exceed the thermal stability threshold of certain flavonoid glycosides, triggering degradation, isomerization, or polymerization reactions that significantly reduce their content [33]. This finding coincides with experimental outcomes reported by Wang et al. [34] on white tea, which further confirms the thermal lability of such compounds.

In summary, bioactive constituents exhibit disparate thermal responses to drying temperatures, reflecting their distinct thermostability and metabolic variation patterns. Therefore, differentiated regulation based on the thermal sensitivity of target components is essential in practical drying processes. A uniform fixed-temperature scheme fails to maximize the retention of all bioactive components. This study provides a theoretical basis for developing precise drying processes that are tailored to the specific functional requirements of *S. vaninii*.

### 4.2. Antioxidant Activity Characteristics of Different Bioactive Constituents and Their Thermal Response Mechanisms

Antioxidant properties and temperature-dependent regulatory patterns of polyphenol, polysaccharide, and flavonoid fractions were clarified via quantitative determination of their scavenging efficiencies against DPPH, superoxide anion, and hydroxyl radicals in the present experiment. Polyphenols exhibited the most potent overall antioxidant capacity, with scavenging efficiencies ranked as DPPH > superoxide anion > hydroxyl radical. Specifically, the scavenging rates for DPPH and superoxide anion increased with rising temperature, peaking at 70 °C. This phenomenon can be rationalized by the fact that high temperatures effectively preserve polyphenol content and maintain the structural integrity of active groups such as phenolic hydroxyls. High-temperature processing can also convert bound polyphenols into free-state forms, which might influence their electron transfer capacity [35]. However, the scavenging capacity of polyphenols for hydroxyl radicals peaks at 55 °C and declines at 70 °C, a phenomenon attributable to thermal degradation of specific heat-labile polyphenol subtypes at higher temperatures [36]. This temperature-dependent accumulation pattern differs from findings in *Sanghuangporus sanghuang*, where flavonoid rather than polysaccharide content was the primary determinant of antioxidant capacity [23]. Such inconsistencies are ascribed to varietal disparities in cultivated specimens’ variable constituent extractability modulated by drying conditions. Polysaccharides exhibited inferior overall antioxidant activity compared to polyphenols and displayed divergent scavenging responses towards different free radicals. The optimal scavenging capacity occurred at 60 °C for DPPH radicals, at 70 °C for superoxide anion radicals, and at 55 °C for hydroxyl radicals. This variation may be associated with temperature-induced changes in polysaccharide molecular weight and conformation, yet further empirical validation is needed to confirm this assumption [37]. Flavonoids exhibit markedly distinct antioxidant activity patterns, with particularly prominent scavenging capacity against superoxide anions. Despite the absence of a maximum flavonoid concentration at 70 °C, their scavenging capacity for DPPH and superoxide anions still increased with an elevated drying temperature. Plausible drivers include selective retention of certain aglycones or hydrolysis of glycosides, both of which await experimental confirmation [38,39]. Additionally, reducing intermediates generated via non-enzymatic browning reactions, such as the Maillard reaction, during dehydration, may contribute to total antioxidant potential [40]. The hydroxyl radical scavenging capacity of the flavonoids peaked at 55 °C, which aligns with the trend in their content. This indicates that this activity depends more directly on the absolute flavonoid content. In summary, the antioxidant capacity of active components depends not only on their concentration but also on chemical transformations, compositional rearrangement, and molecular structural modifications induced by drying temperature. These findings establish theoretical fundamentals for directional improvement of the antioxidant function of *S. vaninii* by regulating the drying temperature.

### 4.3. Structure–Activity Relationship Between Bioactive Constituents and Antioxidant Activity, and the Regulatory Effects of Drying Temperature

Pearson’s correlation analysis provides robust statistical evidence to elucidate the structure–activity relationship between bioactive compounds against antioxidant activity and clarifies the regulatory mechanism mediated by drying temperature. The results show that the contents of both polyphenols and polysaccharides exhibit extremely significant positive correlations with DPPH radical scavenging activity (*r* = 0.96, 0.96) and superoxide anion scavenging capacities (*r* = 0.96, 0.97) (*p* < 0.01). Such correlations implied both categories of compounds possess shared functional moieties (e.g., phenolic hydroxyls or specific sugar units) that can efficiently scavenge these two types of free radicals [41,42]. Furthermore, the polyphenol and polysaccharide contents showed an extremely significant positive correlation (*r* = 0.95), alongside a strong correlation between DPPH and superoxide anion scavenging abilities (*r* = 0.98). These findings demonstrated a potential synergistic relationship between polyphenols and polysaccharides, which may collectively contribute to the core bioactive foundation for eliminating DPPH and superoxide anion radicals. However, correlation cannot conclusively validate synergism, and definitive verification would require combination index studies or isobolographic analysis. In contrast, flavonoid content displayed no significant correlation with any antioxidant indicators, verifying that flavonoids are not the dominant contributor to antioxidant activity in this system. While certain flavonoid monomers possess potent antioxidant potential, their absolute content, structural form (e.g., glycosylation degree), and interactions with other components (e.g., polyphenols and polysaccharides) within complex medicinal fungal systems may influence their ultimate bioactivity [43].

### 4.4. Drying Process Optimization Strategy Based on Cluster Analysis

Six drying temperatures were categorized into two distinct groups via cluster analysis, which lays a comprehensive quality-based foundation for drying parameter optimization. Cluster I (45 °C and 50 °C) corresponds to the ‘low-temperature, slow-speed’ drying mode, featuring inferior overall yields of bioactive constituents and antioxidant activity and unfavorable comprehensive commodity quality. Cluster II (55 °C, 60 °C, 65 °C, and 70 °C) represents a moderate-to-high-temperature, ‘rapid’ drying mode, which significantly enhances the accumulation of bioactive metabolites and improvement of antioxidant function. This classification is consistent with the observed patterns across all indicators and confirms that temperatures at or above 55 °C favor quality preservation of *S. vaninii* fruiting bodies. Specifically, 70 °C is optimal for maximizing bioactive content and antioxidant activity (DPPH and superoxide anion scavenging). However, 55 °C is preferred for targeted enrichment of flavonoids or elevation of hydroxyl radical scavenging activity. Practical production requires balancing drying efficiency, energy costs, and market demands in order to select targeted processes, eliminating uncertainties originating from empirical temperature selection. These findings support the view of Sun et al. [44] that medium-to-high-temperature drying is preferable for exocarpium citri grandis. Collectively, the cluster analysis converts discrete temperature data into practical decision-making references: production should avoid the 45–50 °C range entirely, and individual temperatures inside Cluster II (55–70 °C) can be chosen according to customized quality targets.

### 4.5. Limitations of the Study

While this study elucidates the temperature-dependent accumulation of bioactive constituents and antioxidant activity in *S. vaninii*, several methodological limitations should be noted when interpreting the present findings. First, spectrophotometric methods were only capable of determining the total contents of polysaccharides, flavonoids, and polyphenols without identifying individual monomeric compounds (e.g., specific flavonoid glycosides or polyphenolic monomers). Furthermore, critical structural characteristics are closely associated with antioxidant performance (e.g., molecular weight distribution of polysaccharides, degree of glycosylation of flavonoids). As a result, the observed temperature-induced variations in total content may partially result from altered extractability and structural modification rather than net compound accumulation, which cannot be distinguished by conventional photometric detection. Second, the in vitro antioxidant assays (DPPH, superoxide anion, and hydroxyl radical scavenging) reflect the integrated radical scavenging capacity of total soluble extracts but fail to differentiate synergistic, additive, or antagonistic interactions among different compound classes. Furthermore, the physiological relevance of these chemical-based radical scavenging assays to in vivo antioxidant effects remains to be established. Future research should address these deficiencies by adopting high-performance liquid chromatography (HPLC) or liquid chromatography–mass spectrometry (LC-MS) to quantify individual phenolic and flavonoid compounds. Size-exclusion chromatography is also recommended to profile polysaccharide molecular weight distribution. Additionally, cell-based antioxidant assays or in vivo models would provide more biologically valid evidence of health benefits. Despite these limitations, this study establishes a clear temperature–activity relationship and offers practical guidance for the precise optimization of hot-air drying protocols for cultivated *S. vaninii*.

## 5. Conclusions

This study demonstrates that hot-air drying temperature differentially regulates the bioactive constituents and antioxidant activity of *S. vaninii* fruiting bodies through component-specific mechanisms. Polyphenols and polysaccharides increased progressively from 45 °C to 70 °C, while flavonoid accumulation peaked at 55 °C and declined at higher temperatures. Polyphenols served as the dominant contributors to antioxidant capacity, followed by polysaccharides, whereas flavonoids exhibited a relatively minor influence.

From an industrial perspective, drying at ≥55 °C is recommended to achieve superior overall quality. Specifically, 70 °C is optimal for maximizing polyphenol and polysaccharide contents, as well as DPPH and superoxide anion scavenging capacities, which favors the production of high-antioxidant preparations. In contrast, 55 °C is preferable for flavonoid enrichment or color retention, particularly for applications targeting hydroxyl radical scavenging. These findings provide an evidence-based, temperature-regulated strategy for the post-harvest processing of *S. vaninii*, enabling targeted drying protocols that replace empirical temperature selections with rational choices aligned with specific commercial or functional requirements.

## Figures and Tables

**Table 1:** Effects of hot-air drying temperature on color differences in *S. vaninii* fruiting bodies.

Hot-Air Drying Temperature (°C)	*L*	*a*	*b*	Δ *E*
Fresh	60.04 ± 1.89 b	7.62 ± 0.7 ab	60.73 ± 12.03 c	-
45	62.67 ± 3.72 a	7.53 ± 0.68 bc	63.61 ± 5.76 b	3.9 b
50	62.44 ± 2.48 a	7.78 ± 0.71 a	64.24 ± 4.61 ab	4.26 b
55	62.94 ± 1.74 a	7.57 ± 0.39 b	65.46 ± 3.49 a	5.55 a
60	62.97 ± 2.98 a	7.69 ± 0.5 ab	63.45 ± 2.97 b	4.00 b
65	62.28 ± 2.22 a	7.6 ± 0.56 b	64.5 ± 3.95 ab	4.39 b
70	62.39 ± 3.5 a	7.38 ± 1.12 c	64.21 ± 4.9 ab	4.21 b

Note: Different lowercase letters within the same series denote significant differences (*p* < 0.05). The same applies below.

**Table 2:** Effects of hot-air drying temperatures on the texture of *S. vaninii* fruiting bodies.

Hot-Air Drying Temperature (°C)	Hardness (N)	Elasticity	Cohesion	Adhesiveness (N)	Chewability (N·mm)
Fresh	64.75 ± 15.73 f	3.97 ± 0.73 b	0.46 ± 0.02 a	29.42 ± 6.33 g	113.31 ± 14.37 g
45	197.05 ± 46.34 e	4.18 ± 0.45 a	0.45 ± 0.05 ab	89.00 ± 20.22 f	374.23 ± 104.99 f
50	242.72 ± 115.94 d	3.70 ± 0.54 d	0.41 ± 0.09 c	103.08 ± 61.88 d	397.06 ± 271.84 d
55	274.00 ± 109.3 a	3.81 ± 0.38 c	0.44 ± 0.06 b	124.84 ± 59.55 a	478.09 ± 229.90 a
60	274.90 ± 165.07 a	4.00 ± 0.85 b	0.41 ± 0.07 c	115.30 ± 76.76 b	420.71 ± 235.99 c
65	261.08 ± 112.45 b	3.78 ± 0.46 cd	0.42 ± 0.08 c	113.28 ± 67.07 c	426.69 ± 277.06 b
70	255.40 ± 136.54 c	3.91 ± 0.98 b	0.41 ± 0.20 c	101.21 ± 55.65 e	390.36 ± 205.92 e

Note: Different lowercase letters in the same column indicate significant differences (*p* < 0.05, one-way ANOVA with Tukey’s HSD test).

**Table 3:** Correlation between active substances and antioxidant activity of *S. vaninii*.

Index	Polyphenols	Polysaccharides	Flavonoids	DPPH Radical Scavenging Ability	Superoxide Anion Scavenging Ability	Hydroxyl Radical Scavenging Ability
Polyphenols	1					
Polysaccharides	0.95 **	1				
Flavonoids	0.46	0.27	1			
DPPH radical scavenging ability	0.96 **	0.96 **	0.24	1		
Superoxide anion scavenging ability	0.95 **	0.97 **	0.19	0.98 **	1	
Hydroxyl radical scavenging ability	−0.25	−0.43	0.62	−0.37	−0.42	1

Note: ** significant correlation at 0.01 level.

## Data Availability

The original contributions presented in this study are included in the article. Further inquiries can be directed to the corresponding authors.
